# Association of Adherence to a Mediterranean Diet with Excess Body Mass, Muscle Strength and Physical Performance in Overweight or Obese Adults with or without Type 2 Diabetes: Two Cross-Sectional Studies

**DOI:** 10.3390/healthcare9101255

**Published:** 2021-09-24

**Authors:** Amy Buchanan, Anthony Villani

**Affiliations:** School of Health and Behavioural Sciences, University of the Sunshine Coast, Sunshine Coast, QLD 4556, Australia; agb012@student.usc.edu.au

**Keywords:** Mediterranean diet, adiposity, muscle strength, physical performance, type 2 diabetes mellitus, ageing

## Abstract

Overweight and obesity in older adults is associated with disability and is exacerbated by the presence of type 2 diabetes (T2DM). There is emerging evidence that adherence to a Mediterranean diet (MedDiet) reduces adiposity and attenuates physical disability. These cross-sectional studies explored the associations of adherence to a MedDiet with body mass index (BMI), adiposity, muscle strength, and physical performance in older adults without diabetes and in middle-aged or older adults with T2DM. MedDiet adherence was assessed using the Mediterranean Diet Adherence Screener. Fat mass and percent body fat were assessed by dual energy X-ray absorptiometry. Muscle strength was assessed using hand-grip strength, while physical performance was assessed using the Short Physical Performance Battery and gait speed. A total of *n* = 87 participants with T2DM (T2DM sample: 71.2 ± 8.2 years, BMI: 29.5 ± 5.9) and *n* = 65 participants without diabetes (non-T2DM sample: 68.7 ± 5.6 years, BMI: 33.7 ± 4.9) were included in these analyses. In the T2DM sample, when controlled for age, gender, and appendicular lean mass index, adherence to a MedDiet was inversely associated with BMI, fat mass, and percent body fat. However, this was no longer maintained in the fully adjusted models. Although, adherence to a MedDiet was positively associated with gait speed (β = 0.155; *p* = 0.050) independent of all covariates used. Adherence to a MedDiet may be a suitable dietary strategy for preserving lower body physical function in middle-aged and older adults with T2DM. However, these findings should be further investigated using well-designed randomised controlled trials and prospective cohort studies with a wider range of adherence scores to investigate temporal associations.

## 1. Introduction

With improved life expectancy in an ageing population, the preservation of musculoskeletal health in later life has become increasingly important [1]. Physiological ageing is associated with the gradual loss of lean body mass and the simultaneous increase in adipose tissue and ectopic fat. Consequent reductions in muscle strength and physical performance heighten the vulnerability of middle-aged and older adults to mobility disability, hospitalisations, and loss of independence [2,3], especially in the presence of multiple comorbidities [4] and increasing rates of obesity [5]. Age-related changes in body composition are also unfavourable from a metabolic perspective, with evidence suggesting that additional body fat and reduced muscle mass may contribute to the development of cardiometabolic diseases, including type 2 diabetes mellitus (T2DM) [6,7,8,9]. This is potentially a bidirectional relationship, as the loss of lean body mass, strength, and function is proposed to be accelerated in T2DM [10,11,12] due to underlying pathological mechanisms of insulin resistance, hyperglycaemia, inflammation, and oxidative stress [13]. Consequently, middle-aged and older adults with T2DM are likely to be at a higher risk of adverse musculoskeletal outcomes than those without T2DM, threatening the ability to carry out normal daily activities and negatively affecting quality of life [14,15]. Given that secular trends in diabetes incidence are forecasted to increase in the coming decades [16], strategies to attenuate adiposity and musculoskeletal decline in middle-aged and older adults with T2DM is warranted.

The treatment of T2DM includes a strong focus on weight management by targeting modifiable risk factors including physical activity and nutrition [17]. Of note, resistance training has been associated with improvements in fat mass, lean body mass, and muscle strength in adults with T2DM [18]. In contrast, effective dietary strategies to support musculoskeletal health in older patients with T2DM is less clear. An energy-restricted diet in older adults is currently debated, as resulting energy-restricted weight loss consists of both fat mass and lean body mass [19]. Furthermore, weight regain in older adults is likely to primarily comprise of fat mass [20,21]. This may exacerbate functional and metabolic consequences already present as a result of ageing and T2DM. However, there is evidence to suggest that energy-restricted diets that are higher in protein [22] or combined with physical activity [23,24] may attenuate declines in lean body mass. Evidence for the efficacy of other single-nutrient dietary strategies, including carbohydrate restriction [25], on weight loss in T2DM patients remains equivocal.

There has been growing interest into dietary patterns as a determinant of chronic disease risk over the life course [26], with the Mediterranean diet (MedDiet) one of the most highly investigated for the management of T2DM [27,28]. The traditional MedDiet is a dietary pattern consistent with that typically consumed by populations living in the olive growing regions of the Mediterranean basin before the mid-1960s. Although often described as a ‘diet’ in the research literature, in reality the MedDiet is more correctly recognisable as a ‘whole lifestyle’ approach, which also incorporates time-honoured behaviours, including harvesting, traditional culinary techniques, frugality, and conviviality [29,30,31]. Nevertheless, from a dietary perspective, the MedDiet is often described as a plant-based dietary pattern, consistent with a high intake of vegetables, fruits, nuts, legumes, unprocessed cereals, and daily use of extra-virgin olive oil incorporated into all meals; moderate consumption of fish, shellfish, fermented dairy products (cheese and yoghurt), and wine (typically during meals); and a low or infrequent consumption of meat and meat products, processed cereals, sweets, vegetable oils, and butter [32,33]. As a result of its putative beneficial health effects on cardiometabolic health and healthy ageing, the MedDiet is one of the most widely evaluated dietary patterns in the scientific literature, which includes positive effects on musculoskeletal and functional outcomes in older adults [34,35,36]. Although there is less available evidence supporting this relationship in patients with T2DM, similar findings are beginning to emerge [37,38]. Lastly, adherence to a MedDiet is also inversely associated with adiposity, and in particular, central adiposity [39,40]. However, to the best of our knowledge, the relationship between adherence to a MedDiet and various indices of adiposity and physical function is yet to be thoroughly investigated in vulnerable older populations, including those with T2DM. Therefore, this study explored the independent association between adherence to a MedDiet, body mass index (BMI), and measures of adiposity, strength, and physical performance in community-dwelling, middle-aged and older adults with and without T2DM.

## 2. Materials and Methods

### 2.1. Participants and Recruitment

This study included the use of two separate cross-sectional analyses involving two groups of community-dwelling, middle-aged and older adults recruited from two distinct studies conducted by our group [41,42]. The first study was a 12-week randomised controlled trial with a sample population of otherwise healthy overweight and obese older adults aged ≥60 years, with a BMI ≥ 27 kg/m^2^ [41]. Baseline data from this group will be used for the purposes of the present study, and will be referred to as the non-T2DM sample. The second study was a cross-sectional analysis of adults aged ≥50 years with a confirmed diagnosis of T2DM [42], referred to as the T2DM sample. Exclusion criteria for both groups included the presence of cancer; respiratory, neurological, and renal diseases; physical disability; poor cognition/unable to provide informed consent; and current or recent use of anti-inflammatory drugs corticosteroid agents or sex steroid compounds. All participants were local to the Sunshine Coast, Queensland, Australia, and were recruited using social media platforms, printed flyers, and newspaper advertisements. Both studies were conducted in accordance with the guidelines described in the Declaration of Helsinki and all procedures involving human subjects were approved by the Human Research Ethics Committee (A/16/801) (S/171/123), University of the Sunshine Coast, Queensland, Australia. Written informed consent was obtained from all participants.

### 2.2. Outcome Measures

Detailed information regarding outcome measures described in the present study have been reported elsewhere [41,42]. Briefly, key outcomes included assessment of fat mass, percent body fat, hand-grip strength, and measures of physical performance including gait speed and the Short Physical Performance Battery.

### 2.3. Anthropometry and Body Composition

Body weight was measured using a calibrated digital scale (AND Weighing; HW-KGL, Melbourne, Australia) accurate to the nearest 0.1 kg, with participants barefoot and wearing light clothing. A wall-mounted stadiometer (Holtain Limited, Crymych, United Kingdom) was used to measure height to the nearest 0.1 cm. BMI was calculated using weight (kg) divided by height squared (m^2^). Dual-energy X-ray absorptiometry (DXA) (Lunar iDXA: GE Healthcare, Madison, WI, USA) with GE encore densitometry software (version 16; GE Healthcare) was used to assess whole body composition for estimates of fat mass, total percent body fat, and appendicular lean mass. The sum of lean soft tissue mass in both arms and legs was used to determine total appendicular lean mass. The appendicular lean mass index was calculated using the formula, appendicular lean mass/height^2^ (kg/m^2^) [43]. A single trained technician performed and interpreted all DXA scans in accordance with the Nana et al. protocol [44].

### 2.4. Isometric Hand-Grip Strength

A calibrated hand-held dynamometer (Smedley, Tokyo, Japan) was used to assess isometric hand-grip strength of the dominant hand to the nearest 0.5 kg. Participants were instructed to exert maximal force on the dynamometer while moving the dominant hand from a raised position above the head downwards in a 180-degree arc. One practice trial was permitted followed by three recorded measurements, each separated by a 60-s recovery period. The mean of the three measures was used for analysis.

### 2.5. Measures of Physical Performance

Physical performance was investigated using the Short Physical Performance Battery, a composite measure of three physical tests that stimulate domains of lower extremity function required for activities of daily living, including: (1) balance, (2) lower limb strength, and (3) gait speed [45]. For balance, participants were assessed on their ability to stand in three hierarchal positions with their feet together, semi-tandem, or full-tandem. Lower limb strength was assessed by the completion of five consecutive chair stands without the use of upper extremities. The assessment of gait speed involved participants walking at their usual walking speed without acceleration over a measured distance of 4 metres. Each physical test received a rank from 0–4 and were summed, with higher performance represented by a higher total score. Gait speed was also used as an independent measure of physical performance, with two additional trials timed using a stopwatch. The mean of the two measures was used for analysis.

### 2.6. Adherence to a MedDiet

MedDiet adherence was assessed using the Mediterranean Diet Adherence Screener, a previously validated 14-item tool developed and used in the Prevención con Dieta Mediterránea (PREDIMED) study [46]. Dietary adherence scores are based on pre-defined criteria for consumption and frequency of 12 main dietary components and two food habits related to a Mediterranean dietary pattern. Each item in the tool was dichotomously scored as either 0 or 1, producing a maximum score of 14. Greater adherence to a MedDiet is indicated by a higher score, with categorisation reflecting low (score ≤5), moderate (score 6–9), or high (score ≥10) adherence. For the purpose of the present study, MedDiet adherence was reported as a continuous variable in each of the regression models.

### 2.7. Statistical Analysis

All continuous variables are expressed as means ± standard deviations (SD). The Kolmogorov–Smirnov statistic was used to assess normality of data prior to all tests, and multiple regression diagnostics were performed to ensure assumptions of multicollinearity and homoscedasticity were not violated. Independent samples t tests were used to identify differences in baseline characteristics between the two study groups. Pearson’s correlation coefficients were used independently per study group to identify associations between adherence to a MedDiet and BMI, measures of adiposity, hand-grip strength, and physical performance. Univariable and multivariable linear regression analysis (and 95% CI) was also used to investigate the independent association between adherence to a MedDiet and BMI, measures of adiposity, hand-grip strength, and physical performance using one unadjusted and four adjusted predictor models. Standardised beta-coefficients were used in the univariable and multivariable linear regressions with z-scores for all outcome variables calculated before running each of the regression models. Therefore, the beta-coefficients are interpreted as the change in the predicted value for each of the outcomes based on a standard deviation increase in the Mediterranean Diet Adherence Screener score. Analyses were performed using Statistical Package for the Social Sciences (SPSS) for Windows 26.0 software (IBM Corp., Armonk, NY, USA), with statistical significance set at *p* ≤ 0.05.

## 3. Results

A total of *n* = 87 (male, *n* = 58; female, *n* = 29) middle-aged and older overweight or obese community-dwelling adults with T2DM were included as part of the T2DM study sample. A further *n* = 65 (male, *n* = 22; female, *n* = 43) older overweight or obese community-dwelling adults were included as part of the non-T2DM sample. Participant characteristics according to the two study samples are shown in Table 1. As expected, the non-T2DM sample had a significantly greater body weight (*p* = 0.002), BMI (*p* < 0.001), fat mass (*p* < 0.001), and percent body fat (*p* < 0.001). This sample of participants also had a significantly greater appendicular lean mass index (*p* = 0.050) and faster gait speed (*p* < 0.001). Adherence to a MedDiet was not significantly different between the two study groups.

In the T2DM sample, Pearson’s correlation coefficients showed a small to moderate positive association between adherence to a MedDiet and lower body extremity function including gait speed (*r* = 0.362, *p* < 0.001) and the Short Physical Performance Battery score (*r* = 0.243, *p* = 0.023). Furthermore, independent analysis of the non-T2DM sample found an inverse association between MedDiet adherence and BMI (*r* = −0.274, *p* = 0.027).

Table 2 presents standardised beta-coefficients (and 95% CI) from univariable and multivariable linear regression analysis reported for independent associations between MedDiet adherence and BMI, measures of adiposity, hand-grip strength, and physical performance. When analysing both groups independently, in the T2DM sample, adherence to a MedDiet was inversely associated with BMI (Beta = −0.200; 95% CI: −0.329–0.061; *p* = 0.005), fat mass (Beta = −0.230; 95% CI: −0.385–0.050; *p* = 0.012) and percent body fat (Beta = −0.187; 95% CI: −0.330–0.001; *p* = 0.048) independent of age, gender, and appendicular lean mass index. However, this inverse relationship was not maintained in the fully adjusted model. Nevertheless, a significant positive association was observed between MedDiet adherence and gait speed (Beta = 0.155; 95% CI: 0.006–0.283; *p* = 0.050) independent of all covariates used in the present study. In the non-T2DM sample, adherence to a MedDiet was not associated with adiposity. A significant positive association was observed between MedDiet adherence and gait speed (Beta = 0.265; 95% CI: 0.017–0.519; *p* = 0.037). However, this relationship was not maintained in the fully adjusted model.

## 4. Discussion

To the best of our knowledge, this is one of the first Australian studies to investigate the relationship between MedDiet adherence, adiposity, and physical function in a vulnerable older group at risk of functional decline and mobility disability. We showed that adherence to a MedDiet was not associated with BMI and measures of adiposity in the fully adjusted models used in the present study. However, MedDiet adherence was positively associated with gait speed in the T2DM sample, strengthening the current evidence for the potential role of healthy dietary patterns in attenuating functional decline in vulnerable older adults with chronic disease.

There is previous evidence to support that adherence to healthy dietary patterns, including the MedDiet, is inversely associated with central obesity [47,48]. Specifically, in relation to a diabetic population, results from the present study were somewhat consistent with the overarching literature demonstrating the potential benefits of a MedDiet intervention to support weight loss in overweight and obese adults. In brief, two previously published systematic reviews of randomised controlled trials identified that a MedDiet intervention was effective in reducing BMI [49] and body weight [49,50] in overweight and obese adults across all ages in patients with T2DM. Vitale et al. reported an inverse association between MedDiet adherence and BMI in *n* = 2568 middle-aged and older Italians with T2DM [51]. Moreover, a post-hoc analysis of *n* = 191 participants with T2DM aged 55–80 years from the PREDIMED study also reported similar findings [52]. Specifically, Lasa et al. showed that after a 1-year follow-up, participants randomised to a MedDiet intervention lost significantly more body weight and abdominal fat in comparison to the low-fat control. Although we did not report on measures of abdominal adiposity per se, we observed an inverse association between MedDiet adherence and both fat mass and percent body fat derived from DXA. Importantly, however, this inverse relationship was not maintained when controlled for BMI. Nevertheless, we present novel findings in that few studies have investigated the relationship between MedDiet adherence and measures of adiposity in a sample of middle-aged and older adults with T2DM. As such, our results provide preliminary evidence highlighting the potential benefits of a MedDiet as a dietary strategy to facilitate and promote reductions in adiposity in older adults with diabetes. Furthermore, there is previous evidence to support that adherence to a MedDiet has a myoprotective effect [53], which may attenuate the loss of lean body mass during weight management [54], making it a suitable alternative for older adults vulnerable to adverse changes in body composition and functional decline secondary to energy-restricted weight loss diets.

Several mechanisms may explain the inverse relationship between adherence to a MedDiet and adiposity. A MedDiet is predominantly plant-based and is rich in vitamins, minerals, unsaturated fatty acids, and non-nutritive functional components including polyphenols, phytosterols, and carotenoids [55]. Through the consumption of fruits, vegetables, legumes, wholegrains, nuts, and seeds, a MedDiet is also high in dietary fibre [55,56]. As such, dietary patterns high in fibre have been associated with increased satiation and satiety due to mechanisms of prolonged mastication and the reduction of speed-eating, gastric distention, and the release of cholecystokinin [56]. Furthermore, plant-based foods also have a lower energy-density, thus reducing the risk of excess energy intake and subsequent weight gain [57]. Favourable to the management of T2DM, many of these foods also have a low glycaemic load that dampens the postprandial rise in blood glucose as well as delaying the return of hunger [57,58]. Lastly, while extra virgin olive oil is a fundamental pillar of a traditional MedDiet, research has indicated that the quality of fat is more important than the quantity for the purposes of weight management. Indeed, consumption of unsaturated fats have been associated with increased beta-oxidation, diet-induced thermogenesis, and improved overall energy expenditure [59,60].

Our results further contribute to the growing body of evidence supporting a positive association between adherence to a MedDiet and physical performance in older adults. In agreement with our findings, Shahar et al. reported that greater MedDiet adherence was associated with a faster usual gait speed in older adults from the Health, Aging, and Body Composition (Health ABC) Cohort, with results remaining significant over an 8-year follow-up period [61]. Similar longitudinal results have also been reported in Spanish [62] and Italian [63] cohorts of older adults. However, evidence demonstrating a relationship between MedDiet adherence and hand-grip strength remains inconsistent. In the present study, we showed that adherence to a MedDiet was not associated with hand-grip strength. In contrast, positive relationships have previously been reported in Italian [64], Korean [65], and Portuguese [66] older cohorts. In agreement with findings from the present study, results from the InCHIANTI [63], TRELONG [67], and OSPTRE-FPS [68] studies all showed no relationship between MedDiet adherence and hand-grip strength. Importantly, however, the discrepancy in these findings may indeed be related to the many variations in scoring systems and indices used to quantify adherence to a MedDiet, making comparisons between studies challenging. Specifically, adherence tools such as the Mediterranean Diet Adherence Screener (used in the present study) are based on normative criterion scores, dietary characteristics, and serve sizes that are reflective of a Mediterranean-style diet; in contrast, diet quality indices such as the Mediterranean Diet Score or Alternative Mediterranean Diet Score are dependent on the habitual dietary characteristics of the study population and may not reflect true adherence to a MedDiet pattern [69,70]. Irrespective, few studies have investigated the relationship between MedDiet adherence and functional performance in older adults with T2DM. Tepper et al. [37] previously reported that higher adherence to a MedDiet amongst older Israeli adults with diabetes was associated with lower risk of falls and greater hand-grip strength. However, larger studies that include at-risk populations, including those with T2DM, are needed to confirm these associations and establish temporal relationships. Nevertheless, our results support the potential role of a Mediterranean-style diet to support functional status (e.g., lower body extremity function) as a key component of healthy ageing. Mechanistically, the potential benefits of adherence to a MedDiet and functional status in older adults with chronic disease are likely attributable to the synergistic relationship of nutrients and their role in attenuating processes including inflammation and oxidative stress [53,71]. Low-grade chronic inflammation has been reported in many age-related conditions, including the deterioration of skeletal muscle [72]. Specifically, cytokines including C-reactive protein, interleukin-6, and tumour necrosis factor-α have previously been implicated in the loss of muscle mass, strength, and physical performance in older adults [73,74,75]. A proinflammatory state has also been associated with visceral adiposity and metabolic diseases such as T2DM [72,76], increasing the vulnerability of affected older adults to musculoskeletal and functional decline. However, the high antioxidant capacity of a MedDiet has a key role in modulating signalling pathways involved in the production of inflammatory mediators and reactive oxygen species [77], thereby dampening the inflammatory response, which may aid in preserving muscle integrity. Nevertheless, adherence to a MedDiet and improved functional status may be a marker of healthier lifestyle behaviours and quality of life. Specifically, reverse causation is plausible whereby participants with higher adherence to a MedDiet may also be more physically active and have a better health-related quality of life and, therefore, have greater physical capabilities.

There are several limitations that must be considered in the interpretation of our findings. Firstly, our results may indeed be overstated given that the sample size was relatively small and not generalizable to a wider population of older adults with and without chronic disease. Specifically, an important consideration in the interpretation of our findings is the potential for selection bias at study entry and the heterogeneity in known clinical characteristics between the two study groups. For example, the T2DM sample was significantly older and displayed a slower gait speed. In contrast, the non-T2DM sample had a significantly greater body weight, BMI, and measures of adiposity. Furthermore, despite adjusting for important covariates in our multivariable linear regression, residual confounding cannot be eliminated. For example, we did not control for glycaemia (e.g., HbA1c, glucose, insulin resistance, etc.), blood lipids, and inflammatory markers upon study recruitment, all of which may impact musculoskeletal health and function [78,79,80]. Additional, yet important, confounders requiring consideration include habitual physical activity status, dietary intake (energy and protein in particular), and medication use. Specifically, structured physical activity interventions (particularly resistance exercise) improve body composition (i.e., increase fat-free mass and reduce adiposity), muscle strength, physical performance, and glycaemic control in overweight and obese older adults with and without T2DM [81,82]. In the present study, we also did not consider the use and/or impact of oral hypoglycaemic agents, such as metformin, on changes in body composition, which have been shown in clinical trials to facilitate weight loss [83]. Moreover, adherence scores in the present study were also low to moderate. As of consequence, this limits the generalizability of our results given the low frequency of high adherence scores. Lastly, the cross-sectional design of the present study prevents causality from being determined.

## 5. Conclusions

These cross-sectional analyses showed that adherence to a MedDiet was positively associated with gait speed in middle-aged and older adults with T2DM. Although we are unable to rule out residual confounding, our results do provide novel evidence to suggest that a MedDiet pattern may be a suitable dietary strategy to promote healthy musculoskeletal function in overweight and obese adults with diabetes. However, these findings should be further investigated using well-designed and robust randomised controlled trials and prospective cohort studies with a wide range of adherence scores in order to investigate temporal associations while controlling for important confounders to ascertain the strength of this relationship.

## Figures and Tables

**Table 1:** Participant characteristics according to the two separate (T2DM sample vs. non-T2DM sample) study groups of middle-aged and older adults.

Characteristics	T2DM Sample (*n* = 87)	Non-T2DM Sample (*n* = 65)	*p*
Age (years)	71.2 ± 8.2	68.7 ± 5.6	0.040
Weight (kg)	85.1 ± 19.5	94.3 ± 14.9	0.002
BMI (kg/m^2^)	29.5 ± 5.9	33.7 ± 4.9	<0.001
Fat mass (kg)	31.3 ± 11.1	40.3 ± 9.5	<0.001
Percent body fat (%)	37.9 ± 7.3	44.6 ± 7.3	<0.001
Appendicular lean mass index (kg/m^2^)	7.7 ± 1.5	8.2 ± 1.2	0.050
Hand-grip strength (kg)	33.4 ± 10.8	30.8 ± 10.6	0.138
Gait speed (m/s)	0.9 ± 0.2	1.1 ± 0.2	<0.001
Short Physical Performance Battery score (0–12 points)	10.7 ± 2.1	10.6 ± 1.6	0.742
Mediterranean Diet Adherence Screener score (0–14 points)	5.6 ± 2.3	4.9 ± 2.0	0.070

Data expressed as mean ± SD. T2DM, type 2 diabetes mellitus; BMI, body mass index. *p*-value refers to significant differences between T2DM and non-T2DM study groups.

**Table 2:** Univariable and multivariable linear regression coefficients (and 95% CI) expressing independent associations between adherence to a Mediterranean diet and BMI, measures of adiposity, hand-grip strength, and physical performance by population sample. (standardised beta-coefficient (Beta)) ^a^.

Population Sample	Model	BMI		Fat Mass		Percent Body Fat		Hand-Grip Strength		Gait Speed		Short Physical Performance Battery	
		Beta	*p*	Beta	*p*	Beta	*p*	Beta	*p*	Beta	*p*	Beta	*p*
T2DM	1 ^b^	−0.129 (−0.334, 0.083)	0.235	−0.173 (−0.364, 0.038)	0.110	−0.136 (−0.310, 0.069)	0.209	0.022 (−0.218, 4.806)	0.840	0.362 (0.144, 0.504)	0.001	0.243 (0.037, 0.486)	0.023
	2 ^c^	−0.208 (−0.400, −0.005	0.044	−0.236 (−0.423, −0.023)	0.029	−0.190 (−0.339, 0.069)	0.053	0.093 (−0.308, 3.270)	0.994	0.291 (0.088, 0.437)	0.004	0.161 (−0.035, 0.380)	0.102
	3 ^d^	−0.200 (−0.329, −0.061)	0.005	−0.230 (−0.385, −0.050)	0.012	−0.187 (−0.330, −0.001)	0.048	0.086 (−0.339, 3.412)	0.994	0.291 (0.083, 0.438)	0.004	0.161 (−0.036, 0.381)	0.104
	4 ^e^	−0.036 (−0.085, 0.015)	0.171	−0.009 (−0.059, 0.041)	0.727	−0.039 (−0.032, 0.101)	0.309	0.010 (−0.367, 6.662)	0.936	0.212 (0.006, 0.374)	0.039	0.088 (−0.124, 0.312)	0.393
	5 ^f^	−0.026 (−0.07, 0.026)	0.331	−0.011 (−0.062, 0.040)	0.674	−0.037 (−0.032, 0.102)	0.352	0.005 (−0.312, 4.119)	0.967	0.155 (0.006, 0.283)	0.050	0.040 (−0.207, 0.121)	0.602
Non-T2DM	1 ^b^	−0.274 (−0.464, −0.029	0.027	−0.034 (−4.155, 0.920)	0.790	−0.034 (−0.097, 1.190)	0.788	0.030 (−0.233, 0.297)	0.811	0.227 (0.018, 0.478)	0.068	−0.048 (−0.263, 0.183)	0.707
	2 ^c^	−0.228 (−0.422, 0.012)	0.064	−0.044 (−4.755, 0.985)	0.731	0.045 (−2.038, 2.534)	0.729	0.058 (−0.125, 0.248)	0.512	0.259 (0.010, 0.513)	0.042	−0.042 (−0.271, 0.195)	0.746
	3 ^d^	−0.230 (−0.426, 0.012)	0.064	−0.120 (−0.107, 0.347)	0.249	−0.001 (−0.042, 0.514)	0.094	0.057 (−0.128, 0.247)	0.528	0.265 (0.017–0.519)	0.037	−0.027 (−0.245, 0.197)	0.828
	4 ^e^	−0.230 (−0.426, 0.012)	0.063	−0.078 (−0.185, 0.028)	0.145	−0.039 (−0.028, 0.185)	0.145	0.017 (−0.171, 0.208)	0.847	0.168 (−0.068, 0.407)	0.158	−0.061 (−0.282, 0.172)	0.630
	5 ^f^	−0.117 (−0.31, 0.108)	0.329	−0.088 (−0.188, 0.012)	0.084	−0.088 (−0.012, 0.188)	0.084	0.010 (−0.172, 0.192)	0.913	0.182 (−0.038, 0.406)	0.102	−0.121 (−0.317, 0.099)	0.299

BMI, body mass index; T2DM, type 2 diabetes mellitus. ^a^ Standardised beta coefficient represents the change in a SD-unit increase in the Mediterranean Diet Adherence Screener score per change in outcome measure. ^b^ Non-adjusted model. ^c^ Adjusted for age and gender. ^d^ Adjusted for age, gender, and appendicular lean mass index. ^e^ BMI adjusted for age, gender, appendicular lean mass index, fat mass, and percent body fat; Fat mass adjusted for age, gender, appendicular lean mass index, BMI, and percent body fat; Percent body fat adjusted for age, gender, appendicular lean mass index, BMI, and fat mass; Hand-grip strength adjusted for age, gender, appendicular lean mass index, BMI, fat mass, and percent body fat; Gait speed adjusted for age, gender, appendicular lean mass index, BMI, fat mass, and percent body fat; Short Physical Performance Battery adjusted for age, gender, appendicular lean mass index, BMI, fat mass, and percent body fat. ^f^ BMI adjusted for age, gender, appendicular lean mass index, fat mass, percent body fat, hand-grip strength, gait speed, and Short Physical Performance Battery; Fat mass adjusted for age, gender, appendicular lean mass index, BMI, percent body fat, hand-grip strength, gait speed, and Short Physical Performance Battery; Percent body fat adjusted for age, gender, appendicular lean mass index, BMI, fat mass, hand-grip strength, gait speed, and Short Physical Performance Battery; Hand-grip strength adjusted for age, gender, appendicular lean mass index, BMI, fat mass, percent body fat, gait speed, and Short Physical Performance Battery; Gait speed adjusted for age, gender, appendicular lean mass index, BMI, fat mass, percent body fat, hand-grip strength, and Short Physical Performance Battery; Short Physical Performance Battery adjusted for age, gender, appendicular lean mass index, BMI, fat mass, percent body fat, hand-grip strength, and gait speed.

## Data Availability

The data that support the findings of this study is available from the corresponding author upon reasonable request.
